# Astaxanthin alleviated ethanol-induced liver injury by inhibition of oxidative stress and inflammatory responses via blocking of STAT3 activity

**DOI:** 10.1038/s41598-018-32497-w

**Published:** 2018-09-20

**Authors:** Ji Hye Han, Jung Heun Ju, Yong Sun Lee, Ju Ho Park, In Jun Yeo, Mi Hee Park, Yoon Seok Roh, Sang Bae Han, Jin Tae Hong

**Affiliations:** College of Pharmacy and Medical Research Center, Chungbuk National University, Osongsaengmyeong 1-ro, Osong-eup, Heungdeok-gu, Cheongju, Chungbuk 28160 Republic of Korea

## Abstract

Astaxanthin (AXT) is classified as a xanthophyll carotenoid compound which have broader functions including potent antioxidant, anti-inflammatory and neuroprotective properties. Considerable researches have demonstrated that AXT shows preventive and therapeutic properties against for Diabetes, Osteoarthritis and Rheumatoid Arthritis. However, the protective effect of AXT on liver disease has not yet been reported. In this study, we investigated effects of AXT on ethanol-induced liver injury in chronic plus binge alcohol feeding model. The hepatic steatosis and inflammation induced by ethanol administration were alleviated by AXT. Serum levels of aspartate transaminase and alanine transaminase were decreased in the livers of AXT administrated group. The ethanol-induced expression of cytochrome P450 2E1 (CYP2E1), pro-inflammatory proteins, cytokines, chemokines and reactive oxygen species (ROS) levels were also reduced in the livers of AXT administrated group. Moreover, ethanol-induced infiltration of neutrophils was decreased in the livers of AXT administrated group. Docking model and pull-down assay showed that AXT directly binds to the DNA binding site of STAT3. Moreover, AXT decreased STAT3 phosphorylation in the liver of AXT administration group. Therefore, these results suggest that AXT could prevent ethanol-induced hepatic injury via inhibition of oxidant and inflammatory responses via blocking of STAT3 activity.

## Introduction

Alcoholic liver disease (ALD) is considered to be a major cause of morbidity and mortality worldwide^1,2^. Excessive and chronic alcohol consumption is a leading factor for hepatic injury ranging from simple steatosis to severe forms of liver injury such as steatohepatitis, alcoholic cirrhosis and hepatitis^3^. Ethanol and the products of its metabolism have toxic effects on the liver and induce inflammation and oxidative stress that are key drivers of alcohol-induced liver injury^2^.

Liver is the most targeted organ attacked by oxidative stress^4–6^. Ethanol metabolism triggered Reactive oxygen species (ROS) production during both chronic and acute alcoholism^7^. A variety of cytokines like TNF-α and IL-6 can be also produced in hepatocytes induced by oxidative stress, which might increase inflammation^8^. ROS is toxic to the liver because of DNA damage, mitochondrial dysfunction and lipid peroxidation. ROS could induce by increasing of cytokines and chemokine productions in order to recruit immune cells to the sites of inflammation.

Signal transducer and activator of transcription 3 (STAT3) is a key regulator of various genes involved in inflammatory and oxidative responses^9,10^. Hepatic STAT3 can be activated by various cytokines, growth factors, hormones, and hepatitis viral proteins^11^. Chronic or acute alcohol consumption promotes inflammation in the liver by activation of STAT3. In recent study, ethanol-fed hepatocytes-specific STAT3 knockout (H-STAT3KO) mice produced similar amounts of ROS and pro-inflammatory cytokines such as TNF-α and IL-6 compared with pair-fed mice^9,12^. In addition, IL-6 induced activation of STAT3 and promoted hepatic injury and development of fatty liver^13^.

Astaxanthin (AXT) is ubiquitous in nature, especially in the marine environment, and is found in high amounts in the red-orange pigment of crustacean shells (e.g., crabs, shrimp), salmon, trout and asteroidean. It has been reported that AXT can protect skin from the damaging effects of ultraviolet radiation, ameliorate age-related macular degeneration, protect against chemically induced cancers, increase high-density lipoproteins and enhance the immune system though its anti-oxidant and anti-inflammatory properties^14^. However, its protective effect on alcohol-induced liver injury has not yet been studied. Therefore, we investigated the protective effect of AXT on alcohol-induced liver injury and its mechanism.

## Result

### Astaxanthin alleviated ethanol-induced liver inflammation and liver damages in mice

**Figure 1:**
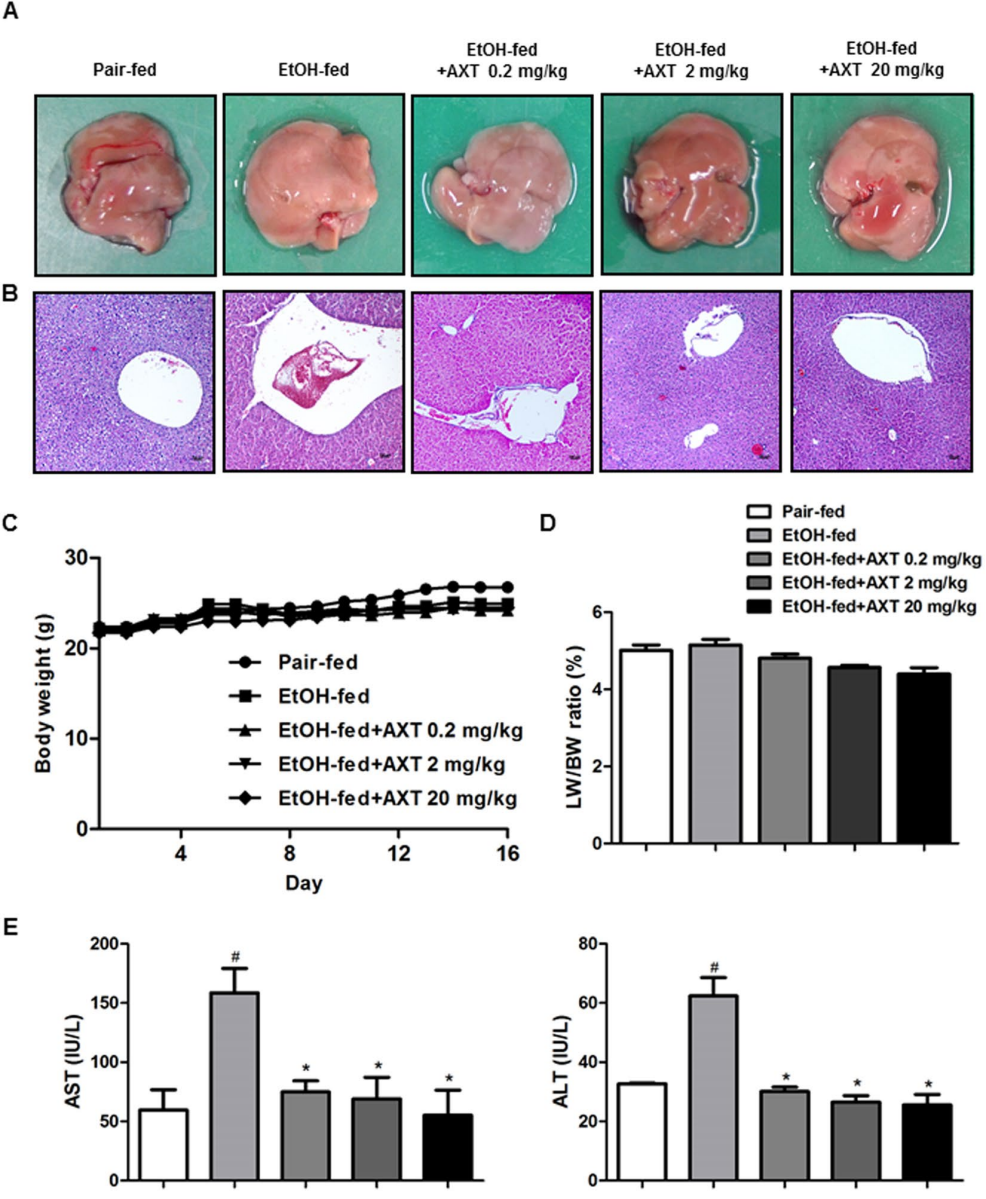
Effects of AXT in chronic-binge ethanol induced alcoholic liver injury in mice. (**A**) Pictures of mouse livers. (**B**) Liver sections of pair-fed mice, ethanol-fed mice and ethanol-fed with AXT (0.2, 2 and 20 mg/kg) mice for 10 days and gavaged a single dose of ethanol (5 g/kg bodyweight) or isocaloric maltodextrin were stained with hematoxylin and eosin (H&E) (scale bars, 100 μm). (**C**) Bodyweight of pair-fed mice, ethanol-fed mice and ethanol-fed with AXT mice during food intake period. (**D**) Ratios of liver to bodyweight, (**E**) serum aspartate transaminase (AST) and alanine transaminase (ALT) levels of pair-fed mice, ethanol-fed mice and ethanol-fed with AXT mice for 10 days and gavaged a single dose of ethanol (5 g/kg bodyweight) or isocaloric maltodextrin. *n* = 8 per group; means ± SD of the mean. ^#^*p* < 0.05, ^##^*p* < 0.01 and ^###^*p* < 0.001 pair-fed *vs*. ethanol-fed, *p < 0.05, **p < 0.01 and ***p < 0.001 ethanol-fed *vs*. ethanol-fed with AXT.

Chronic ethanol exposure induces hepatic steatosis and liver damages. To examine effect of astaxanthin (AXT) on ethanol-induced liver damages in mice, mice were feeding with either ethanol (5% vol/vol) or control liquid diets for 10days in the presence or absence of AXT (0.2, 2 or 20 mg/kg). The liver from chronic-binge ethanol treated group looks rough and was swollen (Fig. 1A). Rate of bodyweight-gain was lower in ethanol-fed mice than in pair-fed mice, but it was higher in ethanol-fed mice than ethanol with AXT-fed mice. The ratio of liver to body weight was increased by chronic-binge ethanol exposure as compared with pair-fed group, which was alleviated by AXT administration (Fig. 1C). Serum levels of aspartate transaminase (AST) and alanine transaminase (ALT) were higher in ethanol-fed mice than pair-fed mice, but these values were decreased in ethanol with AXT-fed mice (Fig. 1D). In addition, histopathology studies revealed fat molecules infiltration, inflammatory cells and necrosis in ethanol-fed mice, but its manifestations were alleviated in ethanol with AXT-fed mice (Fig. 1B).

### Astaxanthin reduced ethanol-induced oxidative stress in the liver of mice

**Figure 2:**
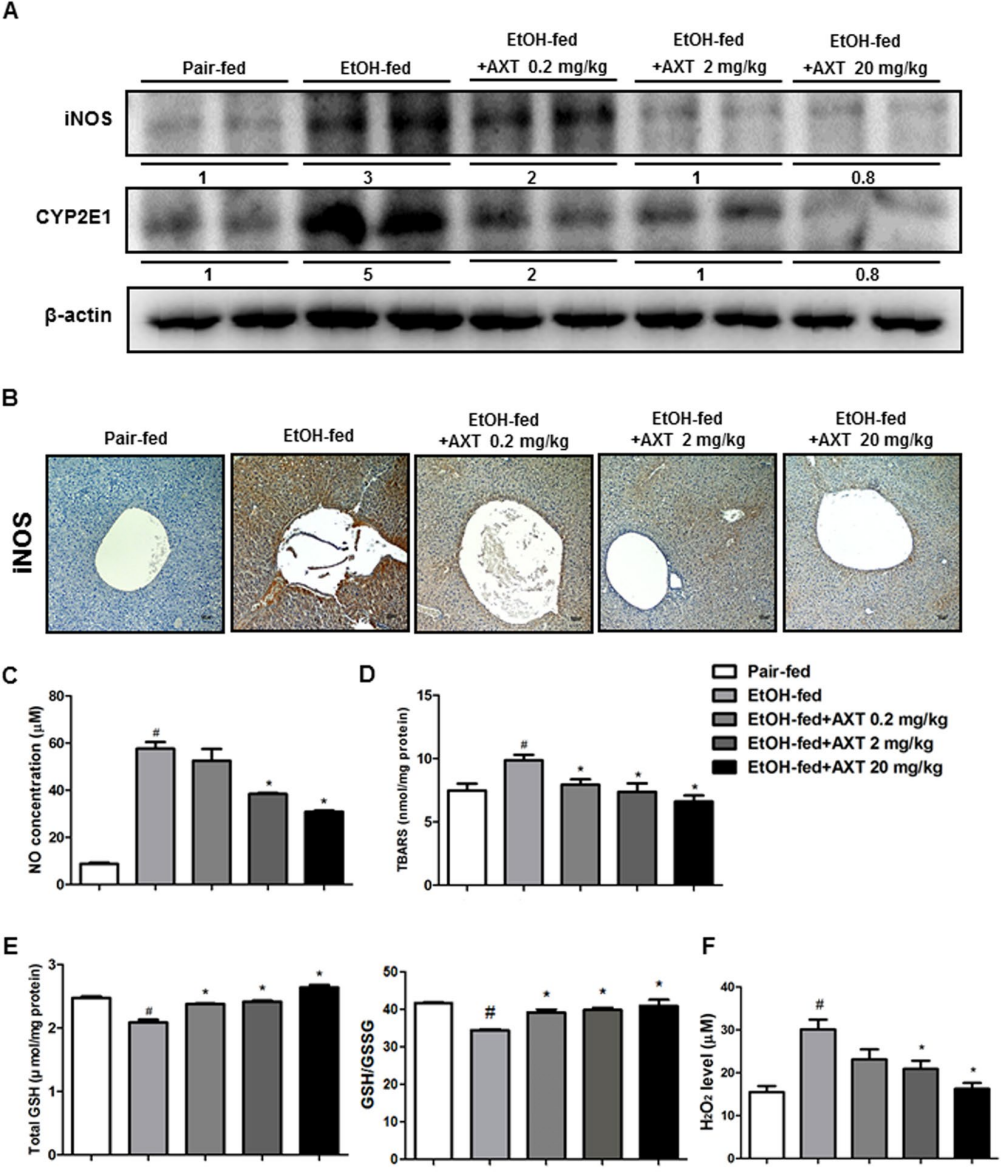
Effects of AXT on ethanol-induced oxidative stress in chronic-binge ethanol induced alcoholic liver injury in mice. (**A**) The expression of iNOS and CYP2E1 were determined in the total protein extracts of mice liver tissues by Western blotting. (**B**) Immunohistochemical analysis of iNOS confirmed in the pair-fed mice, ethanol-fed mice and ethanol-fed with AXT mice (scale bars, 100 μm). (**C**) NO, (**D**) TBARS levels, (**E**) total GSH level and GSH/GSSG ratio and (**F**) hydrogen peroxide level were measured in the pair-fed mice, ethanol-fed mice and ethanol-fed with AXT mice. *n* = 8 per group; means ± SD of the mean. ^#^*p* < 0.05, ^##^*p* < 0.01 and ^###^*p* < 0.001 pair-fed *vs*. ethanol-fed, *p < 0.05, **p < 0.01 and ***p < 0.001 ethanol-fed *vs*. ethanol-fed with AXT.

Because chronic-binge ethanol exposure induces oxidative stress, we determined iNOS and CYP2E1 expression and ROS levels in the livers of pair-fed mice, ethanol-fed mice and ethanol with AXT-fed mice. Ethanol fed increased iNOS and CYP2E1 expression, however AXT administration reduced their expression in a dose dependent manner (Fig. 2A). In addition, immunohistochemistry showed that decrease of the number of iNOS-reactive cells in the liver of ethanol with AXT-fed mice (Fig. 2B). Ethanol fed also elevated NO level, but NO level was decreased in the liver of ethanol with AXT-fed mice (Fig. 2C). The levels of thiobarbituric acid, a marker of lipid peroxidation, was elevated by ethanol, but it was depleted by AXT (Fig. 2D). In addition, the decreased GSH level and GSH/GSSG ratio were recovered by AXT (Fig. 2E). Hydrogen peroxide level was also elevated by ethanol, but it was decreased by AXT (Fig. 2F).

### Astaxanthin decreased ethanol-induced hepatic inflammation in the liver of mice

**Figure 3:**
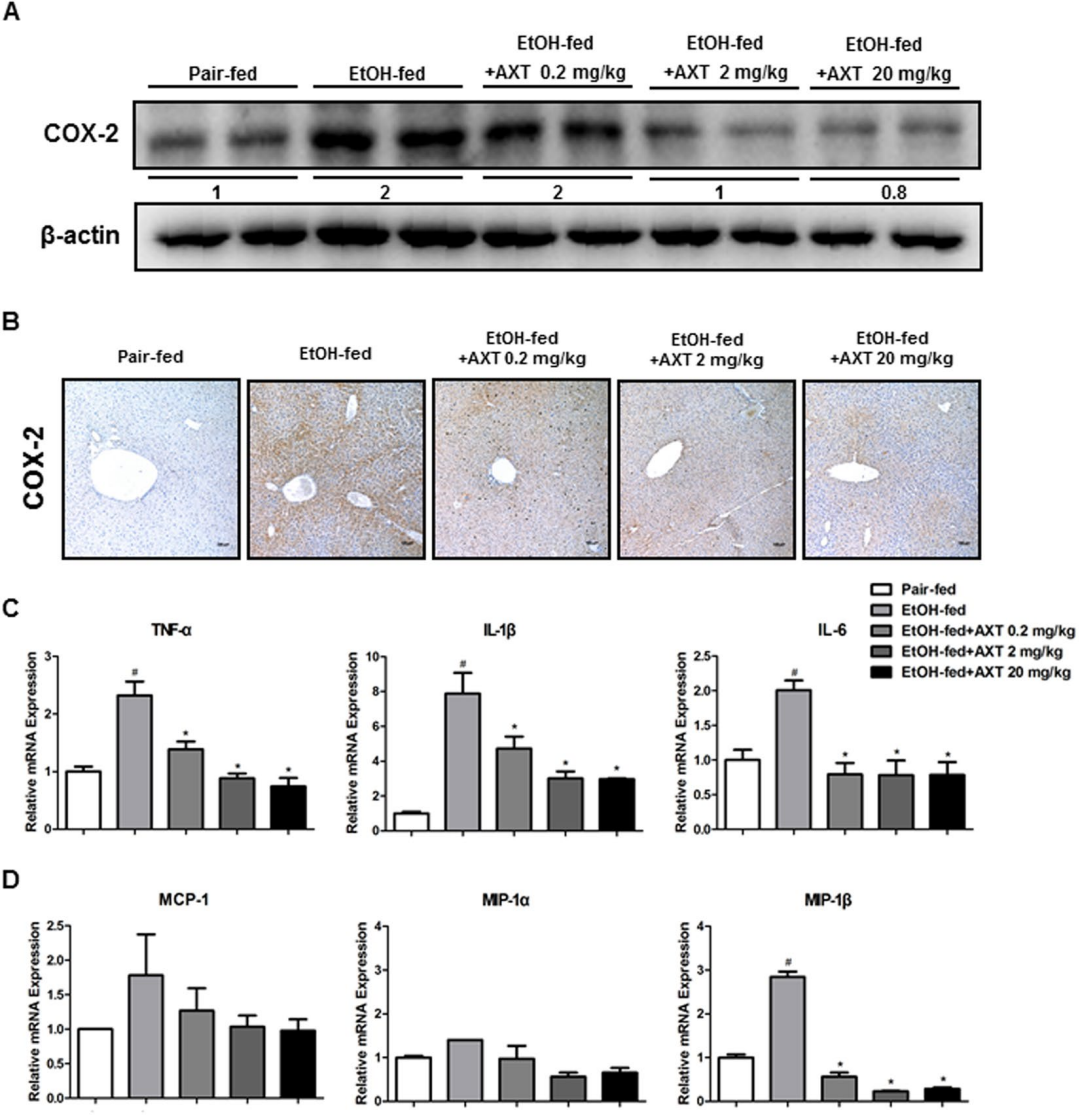
Effects of AXT on inflammatory responses in chronic-binge ethanol induced alcoholic liver injury in mice. (**A**) The expression of COX-2 were determined in the total protein extracts of mice liver tissues by Western blotting. (**B**) Immunohistochemical analysis of COX-2 confirmed in the pair-fed mice, ethanol-fed mice and ethanol-fed with AXT mice (scale bars, 100 μm). (**C**) mRNA expression levels of pro-inflammatory cytokines such as IL-6, TNF-α and IL-1β and (**D**) chemokines such as MCP-1, MIP-1α and MIP-1β in the pair-fed mice, ethanol-fed mice and ethanol-fed with AXT mice. *n* = 8 per group; means ± SD of the mean. ^#^*p* < 0.05, ^##^*p* < 0.01 and ^###^*p* < 0.001 pair-fed *vs*. ethanol-fed, *p < 0.05, **p < 0.01 and ***p < 0.001 ethanol-fed *vs*. ethanol-fed with AXT.

Expression of COX-2 and production of pro-inflammatory cytokines and chemokines are implicated in alcoholic liver diseases. Thus, we determined these factors in the liver. Ethanol-fed increased COX-2 expression but, COX-2 expression was lower in the livers of ethanol with AXT-fed mice (Fig. 3A). We also performed immunohistochemical staining for COX-2. The number of COX-2-reactive cells was lower in the liver of ethanol with AXT-fed mice (Fig. 3B). Furthermore, the expression of a variety of pro-inflammatory cytokines and chemokines in the liver was examined by real-time PCR. Ethanol with AXT-fed mice showed a significant decrease in the levels of pro-inflammatory cytokines such as IL-6, TNF-α and IL-1β, and chemokines such as MCP-1 and MIP-1β (Fig. 3C,D).

### Astaxanthin reduced neutrophils recruitment in the liver of mice

**Figure 4:**
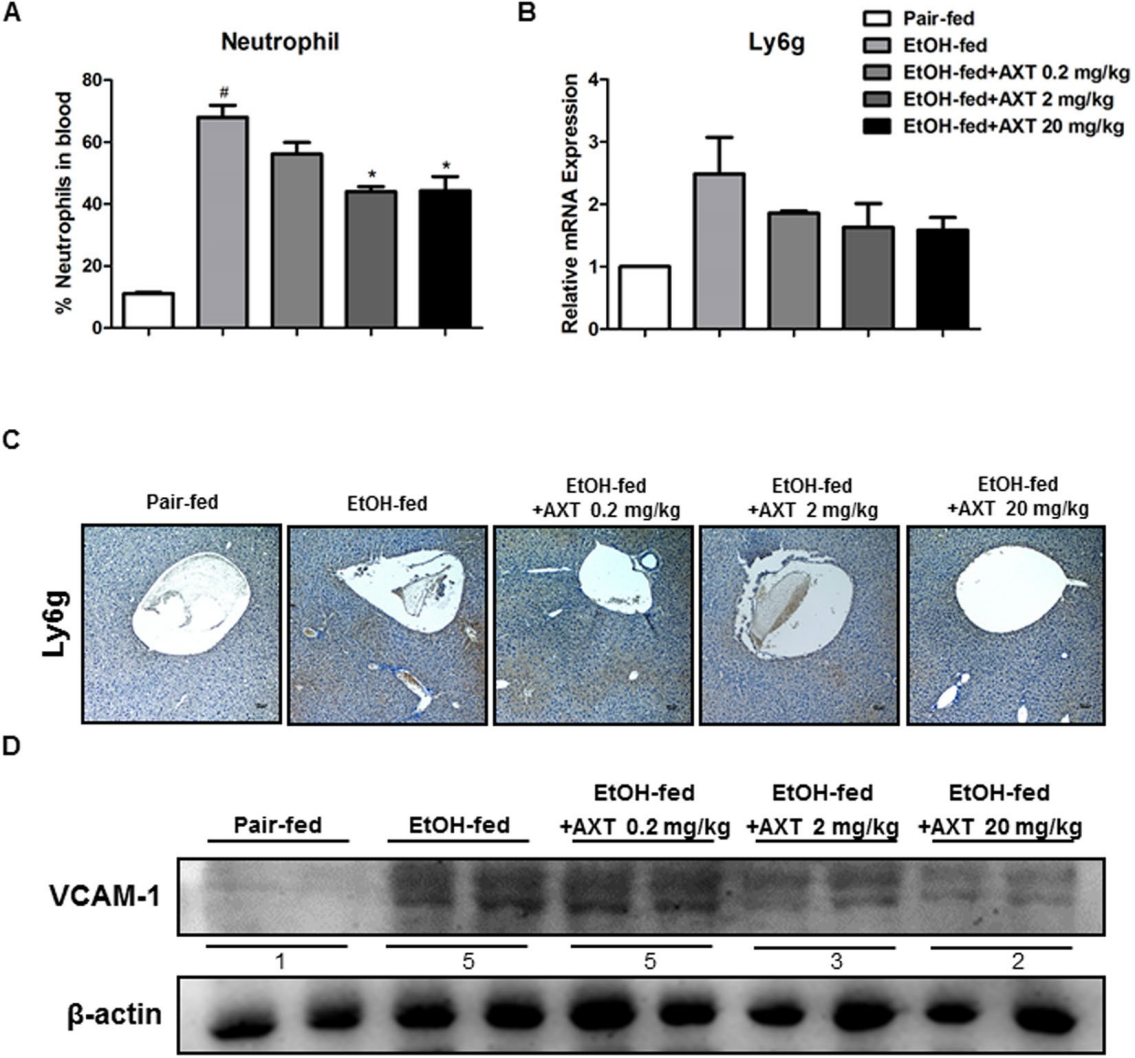
Effects of AXT on infiltration of immune cells in chronic-binge ethanol induced alcoholic liver injury in mice. (**A**) Neutrophil levels in blood of pair-fed mice, ethanol-fed mice and ethanol-fed with AXT mice for 10 days and gavaged a single dose of ethanol (5 g/kg bodyweight) or isocaloric maltodextrin. (**B**) mRNA expression levels of Ly6g, a marker of neutrophil, in the pair-fed mice, ethanol-fed mice and ethanol-fed with AXT mice. (**C**) Immunohistochemical analysis of Ly6g confirmed in the pair-fed mice, ethanol-fed mice and ethanol-fed with AXT mice (scale bars, 100 μm). (**D**) The expression of VCAM-1 were determined in the total protein extracts of mice liver tissues by Western blotting. *n* = 8 per group; means ± SD of the mean. ^#^*p* < 0.05, ^##^*p* < 0.01 and ^###^*p* < 0.001 pair-fed *vs*. ethanol-fed, *p < 0.05, **p < 0.01 and ***p < 0.001 ethanol-fed *vs*. ethanol-fed with AXT.

Neutrophil infiltration is a hallmark of alcoholic hepatitis^15,16^. Neutrophil infiltration likely contributes to hepatocellular damage, possibly by killing hepatocytes through production of ROS^15^. To investigate whether the inhibition of ethanol-induced hepatotoxicity in the ethanol with AXT-fed mice was related to neutrophil infiltration, we analyzed the distribution of neutrophils in liver tissue. Blood level of neutrophils was elevated by ethanol, but down-regulated in ethanol with AXT-fed mice (Fig. 4A). mRNA expression of Ly6G (a neutrophil marker) was also decreased in ethanol with AXT-fed mice (Fig. 4B). In addition, immunohistochemistry of Ly6G also demonstrated level of hepatic neutrophil infiltration was lower in ethanol with AXT-fed mice (Fig. 4C). The expression vascular cell adhesion molecule (VCAM-1) that controlled neutrophil recruitment was significantly increased in ethanol-fed mice but decreased in ethanol with AXT-fed mice (Fig. 4D).

### Astaxanthin reduced the phosphorylation of STAT3 in the liver of mice

**Figure 5:**
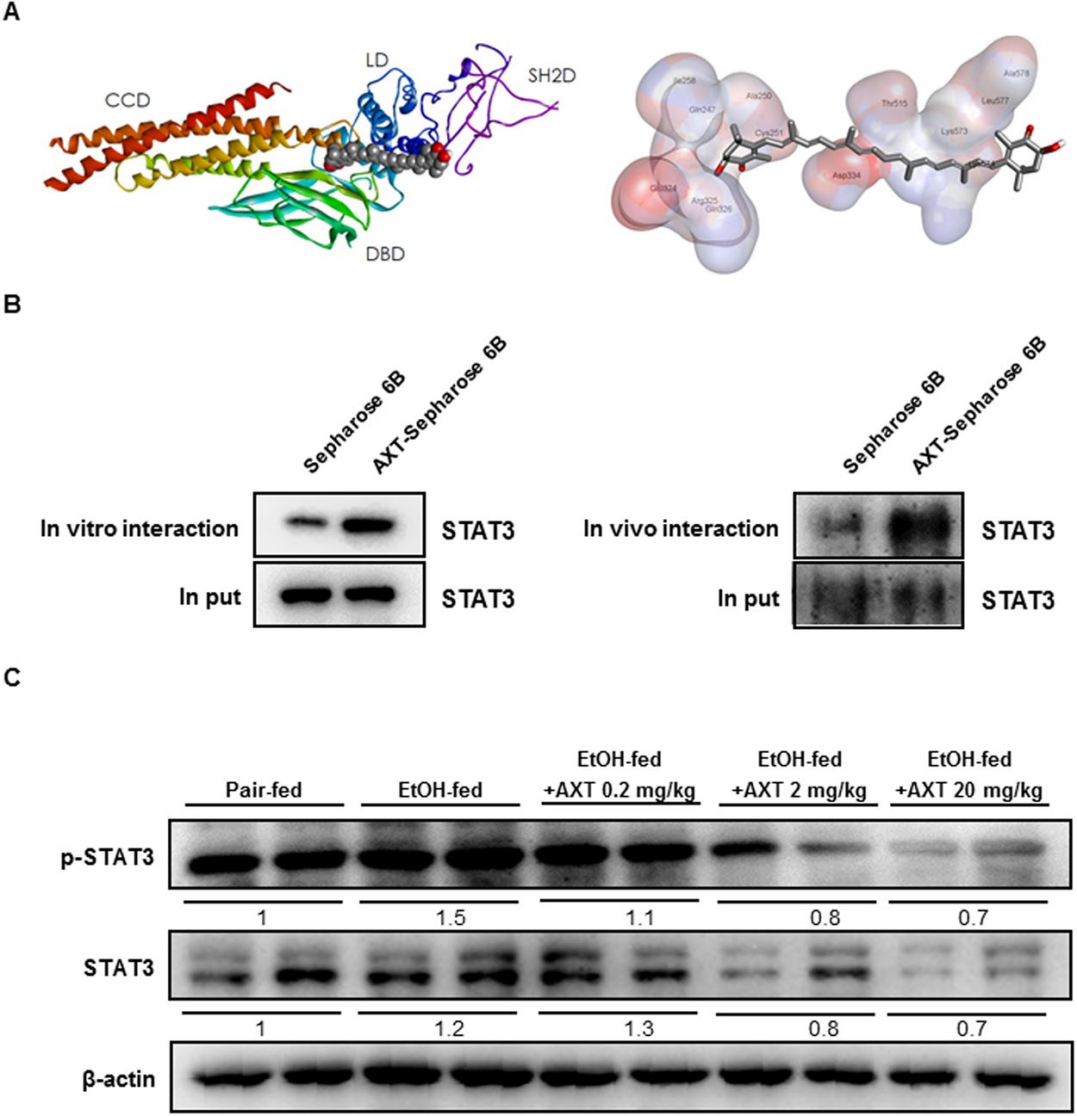
A key role of STAT3 by binding AXT in chronic-binge ethanol induced alcoholic liver injury in mice. (**A**) Docking model of AXT bound with STAT3. (**B**) The expression of STAT3 on presence or absence AXT *in vitro* and *in vivo*. (**C**) The expression of STAT3 and p-STAT3 were determined in the total protein extracts of mice liver tissues by Western blotting.

To clear the STAT3 involvement in the blocking effect of AXT on the inflammatory protein expression and ROS generation, we determined the interaction between AXT and STAT3. The result of the docking studies showed that AXT binds with Lys 574 residue in STAT3 catalytic site and the binding affinity for best binding mode was −9.0 kcal/mol (Fig. 5A). The expression of STAT3 protein that is cell lysates from HEK 293 cells transfected with STAT3 and is expressed in cell-free system in AXT-Sepharose 6B bead was higher than in Sepharose 6B bead (Fig. 5B). Furthermore, ethanol-induced p-STAT3 expression was lowered in ethanol with AXT-fed mice than ethanol-fed mice (Fig. 5C).

## Discussion

Studies conducted during the past few years that astaxanthin (AXT) plays a key role in anti-oxidant and anti-inflammatory responses^14^. In the present study, we demonstrated AXT alleviated ethanol-induced oxidative stress, liver inflammation and thus liver injury by inhibition of STAT3 activity.

Ethanol exposure significantly induced hepatic injury in the livers of ethanol-fed mice, but it was alleviated in ethanol with AXT-fed mice. AXT administration reduced ethanol-induced AST and ALT levels accompanied by increased of liver lipid droplet. In the liver tissue of ethanol-fed mice, many inflammatory cells activated and lipid droplets were revealed by pro-inflammatory cytokines and COX-2 expression. However, in the liver tissue of ethanol with AXT-fed mice, inflammatory cells were decreased and lipid droplets were smaller. Even though we didn’t directly compare the effectiveness of AXT to other compounds showing protective effects for hepatic injury, AXT may more effective to other well-known compounds such as silymarin and green tea in terms of dose and treatment period and protective values. AXT administration (0.2 mg/kg) for 10 days completely alleviated the levels of AST and ALT similar to pair-fed mice, whereas an Korean Red Ginseng administration (250 mg/kg) for 10 days in ethanol-fed mice recovered about half^17^, green tea administration (7 g/L) for 5 weeks in ethanol-fed rat recovered about one-third^18^. Thus, the present data indicated that AXT could be more potent hepatic protective agent for ethanol-induced liver damages.

ALD is closely associated with oxidative stress^19,20^ and the release of pro-inflammatory cytokines/chemokines, particularly TNF-a and IL-6 and NO that contribute hepatocyte dysfunction^20–22^. Excessive ethanol exposure-induced CYP2E1 and iNOS expression leads to oxidative stress by the generation of ROS and increase in NO production in the hepatocytes of liver^23,24^. The expression of CYP2E1 and iNOS were decreased in in ethanol with AXT-fed mice. In the liver tissue of ethanol with AXT-fed mice, iNOS-reactive cells were decreased. In addition, the production of NO was also decreased in ethanol with AXT-fed mice. The GSH depletion resulted in the inhibition of oxidative stress^25^. In present study, total GSH levels were depleted in ethanol with AXT-fed mice. Moreover, level of lipid peroxidation in ethanol with AXT-fed mice was also decreased compared with ethanol-fed mice. These data suggest that reduced oxidative damages could be associated with hepatic protective effect of AXT.

Ethanol-induced liver damage is involved in inflammatory responses. IL-6, a major pro-inflammatory cytokine, is elevated by ethanol consumption and is closely associated with ALD^26^. It is worthy to note that patients with severe alcoholic hepatitis who do not respond to medical treatment have low hepatic expression of TNF and IL-6^1^. In our study, the level of IL-6 was significantly reduced in the liver of ethanol with AXT-fed mice. Furthermore, other inflammatory cytokines such as TNF-α and IL-1β and chemokines such as MCP-1 and MIP-1β were also down-regulated in the liver of ethanol with AXT-fed mice. COX-2 is induced by pro-inflammatory cytokines and oxidant stress^27^. iNOS expression is increased by excessive ethanol consumption and is also induced by pro-inflammatory cytokines^24^. In the present study, ethanol-induced COX-2 and iNOS expression were decreased in the liver of ethanol with AXT-fed mice. Therefore, these results suggest that AXT alleviated ethanol-induced pro-inflammatory responses, and thus ameliorated ethanol-induced liver damages.

Neutrophils accumulated in the hepatic microvasculature can extravasate into the hepatic parenchyma if they receive appropriate signals from previously sensitized or distressed cells^28,29^. Infiltration of a large number of neutrophils is a very prominent feature of alcoholic hepatitis^28–30^. The number of neutrophils in blood was elevated in ethanol-fed mice, but it was decreased in ethanol with AXT-fed mice. In addition, the mRNA expression of Ly6g and neutrophils in liver tissue was also reduced in ethanol with AXT-fed mice. Neutrophil recruitment is mediated by a multistep adhesion cascade that involves multiple adhesion molecules and their ligands, which are expressed on endothelial cells (ECs) neutrophils including ICAM-1 and VCAM-1^16^. The expression of VCAM-1 were decreased in ethanol with AXT-fed mice. Thus, reduced neutrophil recruitment could be associated with the reducing effect of AXT in the ethanol-mediated inflammatory associated liver damages.

STAT3 plays important function of in the hepatic inflammation during ALD^31^. Many studies have showed that STAT3 is a transcription factor that is activated by a variety of factors, including cytokines, growth factors, hormones, and hepatitis viral proteins in the liver^11^. Ethanol-fed H-STAT3KO mice produced similar amounts of ROS and pro-inflammatory cytokines such as TNF-α and IL-6 compared with pair-fed mice^9^. Several studies demonstrated that inhibition of STAT3 could be associated with reduction of liver damages. STAT3 was activated in aldehyde dehydrogenase 2 deficiency mice which were more prone to ethanol^32^. Compounds from natural resources such as Anthocyanins and Kavalactone desmethoxyyangonin attenuated ethanol or LPS-induced liver damages through inhibition of STAT3^33,34^. Moreover, inhibition of STAT3 was associated with decrease effectiveness of several other disease. Stevia and Stevioside protected cisplatin nephrotoxicity by inhibition of STAT3^35^. Corydalis hendersonii Hemsl prevented myocardial injury by attenuating inflammation and fibrosis via STAT3 inhibition^36^. These results indicated that STAT3 could mediate ethanol-induced inflammatory responses in the liver. In our study, docking model shows AXT was directly binding with STAT3. We also showed AXT was binding with STAT3 protein *in vivo* and *in vitro*. Furthermore, ethanol-induced STAT3 phosphorylation was decreased in ethanol with AXT-fed mice than in ethanol-fed mice.

In summary, our results suggest that AXT protects against ethanol-induced liver injury. This effect may result from a reduction in oxidative stress and inflammatory responses by blocking STAT3 activity.

## Materials and Methods

### Animals experiment and housing condition

Male C57BL/6 (8 weeks old) mice were purchased from DBL (Eumsung, Korea). The experiment was performed in accordance with the guidelines proscribed by the Chungbuk National University Animal Care Committee (CBNUA-929-16-01). The mice were acclimatized to the laboratory environment, maintained at 22 ± 1 °C and relative humidity of 55 ± 10%, with 12 h light-dark cycles throughout the experiment. All mice were fed a standard laboratory chow diet *ad libitum*. All mice were randomly divided into the following five groups (*n* = *8*/group): control group, ethanol group (5% vol/vol), ethanol + AXT 0.2 mg/kg group, ethanol + AXT 2 mg/kg group, ethanol + AXT 20 mg/kg group. The mice from AXT groups were daily administrated AXT that dissolved in olive oil for 10 days by oral gavage. On the morning of 11^th^ day, acute administration was performed and sacrificed 9 h post gavage.

### Chronic plus binge alcohol feeding model

Male C57BL/6 mice (8 weeks old) administered a Lieber/DeCarli Regular Liquid Diet-Control (Dyets, Cat # 710027) for 5 days with *ad libitum*. Following acclimation, mice were feeding *ad libitum* with a Lieber/DeCarli Regular Liquid Diet-Ethanol (Dyets, Cat # 710260) containing 5% (vol/vol) ethanol or pair-fed a Lieber/DeCarli Regular Liquid Diet-Control for 10 days. On the morning of 11^th^ day, mice were gavaged with a single dose of ethanol (5 g/kg bodyweight) or maltodextrin, respectively, and were sacrificed 9 h later^37^.

### Measurements of serum aspartate transaminase and alanine transaminase

Blood was collected at 9 h after the ethanol administration. Serum was separated by centrifugation at 3000 rpm for 8 min at 21 °C. Serum aspartate transaminase (AST) and alanine transaminase (ALT) were measured using a biochemical analyzer (AU480, Beckman Coulter, CA, USA).

### Histopathological analysis

For histological processing, liver tissues were fixed in 4% formalin solution. Then, liver tissues were embedded in paraffin. Specimens were sectioned 4 μ and stained with hematoxylin and eosin stain (H&E), then observed with a light microscope (Nikon, Tokyo, Japan).

### Western blot analysis

Homogenized liver tissues were lysed by protein extraction solution (PRO-PREP, iNtRON, Sungnam, Korea) and the total protein concentration was determined using the Bradford reagent (Bio-Rad, Hercules, CA, USA). 40 μg of extracted protein were separated by SDS/PAGE and transferred to Immobilon® PVDF membranes (Millipore, Bedford, MA, USA). The membrane was blocked with 5% dried skimmed milk for 1 h at room temperature, followed by incubation with specific primary antibodies for overnight at 4 °C. The membranes were washed with Tris-buffered saline containing 0.05% Tween-20 (TBST) and incubated with diluted horse radish peroxidase-conjugated secondary antibodies for 1 h at room temperature. After washes, binding of antibodies to the PVDF membrane was detected using the Immobilon Western Chemilum HRP substrate (Millipore, Bedford, MA, USA). The band intensities were measured using the Fusion FX 7 image acquisition system (Vilber Lourmat, Eberhardzell, Germany). Specific primary antibodies were purchased from Santa Cruz Biotechnology (p-STAT3, STAT3 and β-actin; Dallas, TX, USA), Cell signaling Technology (iNOS and COX-2; Trask Lane, Danvers, MA, USA) and Abcam (CYP2E1; Cambridge, MA, USA). Secondary antibodies were purchased from Santa Cruz Biotechnology (anti-mouse and anti-rabbit; Dallas, TX, USA).

### Immunohistochemistry

Paraffin-embedded ethanol-induced liver tissue sections were blocked for 60 min with 2% normal horse serum contained blocking solution diluted in 1X PBS. The sections were then blotted and incubated with specific primary antibodies in blocking solution for overnight at 4 °C. And then, the sections were washed three times for 10 min each in PBS and incubated in biotinylated anti-mouse, rabbit, goat antibody for 90 min. The sections were washed three times for 10 min each in PBS, followed by formation of the avidinbiotin-peroxidase complex (Vector Laboratories, Burlingame, CA, USA). The slides were washed and the peroxidase reaction developed with diaminobenzidine and peroxide and then counter-stained with hematoxylin, mounted in Cytoseal XYL (Thermo Fisher Scientific, Waltham, MA, USA) and evaluated on a light microscope (Nikon, Tokyo, Japan). Specific primary antibodies were purchased from Cell signaling Technology (COX-2; Trask Lane, Danvers, MA, USA) and Abcam (iNOS; Cambridge, MA, USA).

### RNA isolation and quantitative real-time RT-PCR

Total RNA from liver tissues were extracted by RiboEx^TM^ Total RNA isolation solution (GeneAll Biotechnology, Seoul, Korea) and cDNA was synthesized using High Capacity RNA-to-cDNA kit (Applied Biosystems, Foster City, CA, USA). Quantitative real-time RT-PCR was performed on a 7500 real-time PCR system (Applied Biosystems, Foster City, CA, USA) for custom-designed primers and β-actin was used for house-keeping control using QuantiNova SYBR Green PCR kit (Qiagen, Hilden, Germany). Cycling conditions consisted of a denaturation of 5 s at 95 °C and a combined annealing/extension of 10 s at 60 °C followed by 40 cycles. The values obtained for the target gene expression were normalized to β-actin and quantified relative to the expression in control samples.

### Molecular modeling

The stereochemical structure of STAT3 was used for the docking study. Docking studies between AXT and STAT3 were performed using AutoDock VINA (Trott and Olson, 2010). Starting from the co-crystallized complexes, the STAT3 monomer chain (STAT3 from 3CWG), AXT (AXT from Chem3D) for docking were prepared using AutoDock Tools. Docking experiments were performed at various exhaustiveness values of the default, 16, 24, 32, 40 and 60. Molecular graphics for the best binding model was generated using Discovery Studio Visualizer 2.0.

### Pull down assay

AXT was conjugated with Epoxy-activated Sepharose 6B (GE Healthcare Korea, Seoul, Korea). Briefly, AXT (1 mg) was dissolved in 1 ml coupling buffer (0.1 M NaHCO_3_ and 0.5 M NaCl, pH 6.0). The Epoxy-activated Sepharose 6B beads (0.1 g) were swelled and washed in 1 mM HCl on a sintered glass filter, then washed with the coupling buffer. Epoxy-activated Sepharose 6B beads were added to the AXT-containing coupling buffer and rotated at 4 °C for overnignt. The control unconjugated Sepharose 6B beads were prepared as described above in the absence of AXT. After washing, unoccupied binding sites were blocked with blocking buffer (0.1 M Tris-HCl, pH 8.0) at room temperature for 2 h. The AXT-conjugated Sepharose 6B was washed with three cycles of alternating pH wash buffers (buffer 1, 0.1 M acetate and 0.5 M NaCl, pH 4.0; buffer 2, 0.1 M Tris-HCl and 0.5 M NaCl, pH 8.0). AXT-conjugated beads were then equilibrated with a binding buffer (0.05 M Tris-HCl and 0.15 M NaCl, pH 7.5). To demonstrate binding AXT and STAT3 *in vitro* and *in vivo*, STAT3 protein was expressed in two ways; following cell-free system and transfection. HEK293 cells were transfected with STAT3 DNA using Lipofectamine® 3000 (Invitrogen, Waltham, MA, USA) in Opti-MEM, following the manufacturer’s protocol. STAT3 DNA was translated to protein using AccuRapid™ Protein Expression Kit (Bioneer, Alameda, CA, USA) following the manufacturer’s protocol. The STAT3 protein was mixed with AXT-conjugated Sepharose 6B or Sepharose 6B and incubated at 4 °C for overnight. The beads were then washed three times with TBST. The bound proteins were eluted with SDS loading buffer and were separated by SDS/PAGE followed by immunoblotting with antibody against STAT3 (Santa Cruz Biotechnology, Dallas, TX, USA).

### Nitro oxide and oxidative stress assay

Nitro oxides (NO) were measured according to the manufacturer’s protocol (iNtRON, Sungnam, Korea). Hydrogen peroxides assay was performed as described in the manufacturer’s protocol (Cell Biolabs, San Diego, CA, USA). Hepatic levels of reduced glutathione (GSH) and oxidized glutathione (GSSG) were measured using GSH/GSSG Ratio Detection Assay Kit (Abcam, Cambridge, MA, USA). Lipid peroxidation was measured by determining the generation of malondialdehyde (MDA; TBARS Assay kit, Cayman, Ann Arbor, MI, USA).

### Statistical analysis

The data were analyzed using the GraphPad Prism 4 version 4.03 software (Graph-Pad Software, La Jolla, CA). Data are presented as mean ± SD. The differences in all data were assessed by one-way analysis of variance. When the P value in the analysis of variance test indicated statistical significance, the differences were assessed by the Tukey’s test. A value of p ≤ 0.05 was considered to be statistically significant.

## Electronic supplementary material

Supplymentary information (Western blot whole blot)
