# Supplementary material for: Astaxanthin alleviated ethanol-induced liver injury by inhibition of oxidative stress and inflammatory responses via blocking of STAT3 activity
Source: Sci Rep. 2018 Sep 20;8:14090. doi: 10.1038/s41598-018-32497-w (PMC6148091; doi:10.1038/s41598-018-32497-w)

Astaxanthin alleviated ethanol-induced liver injury by inhibition of oxidative stress and inflammatory responses via blocking of STAT3 activity

Ji Hye Han[1], Jung Heun Ju[1], Yong Sun Lee[1], Ju Ho Park[1], In Jun Yeo[1], Mi Hee Park[1], Yoon Seok Roh[1], Sang Bae Han[1], Jin Tae Hong[1*]

[1]College of Pharmacy and Medical Research Center, Chungbuk National University, Osongsaengmyeong 1-ro, Osong-eup, Heungdeok-gu, Cheongju, Chungbuk, 28160, Republic of Korea

*Correspondence : Dr. Jin Tae Hong (jinthong@chungbuk.ac.kr), College of Pharmacy and Medical Research Center, Chungbuk National University, Osongsaengmyeong 1-ro, Osong-eup, Heungdeok-gu, Cheongju, Chungbuk, 28160, Republic of Korea, Tel: +82-043-261-2813, Fax: +82-043-268-2732

Full images of electrophoretic blot in figure 2

iNOS

75 kDa —

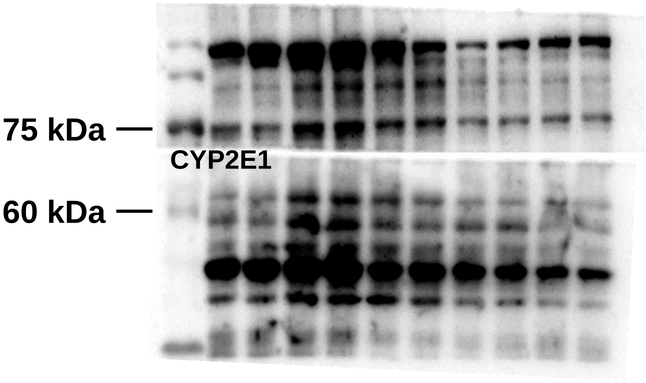

CYP2E1

60 kDa —

β-actin

75 kDa —

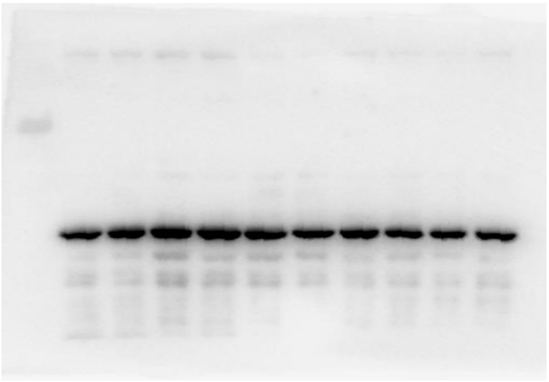

Full images of electrophoretic blot in figure 3

COX-2

75 kDa —

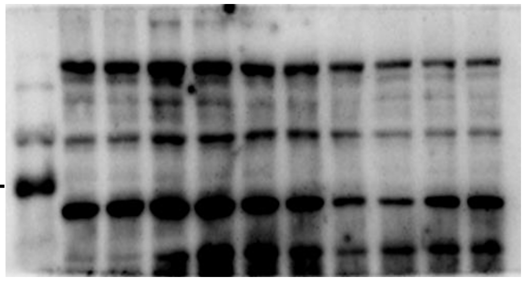

β-actin

75 kDa —

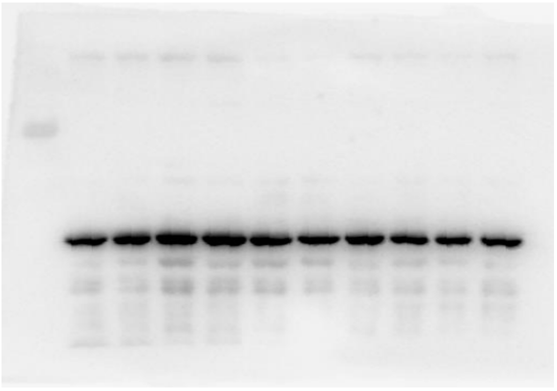

Full images of electrophoretic blot in figure 4

VCAM-1

100 kDa —

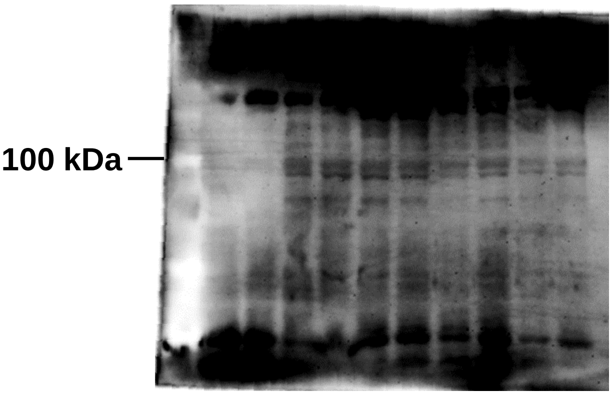

β-actin

45 kDa —

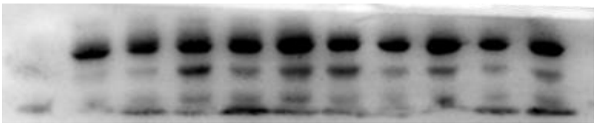

Full images of electrophoretic blot in figure 5

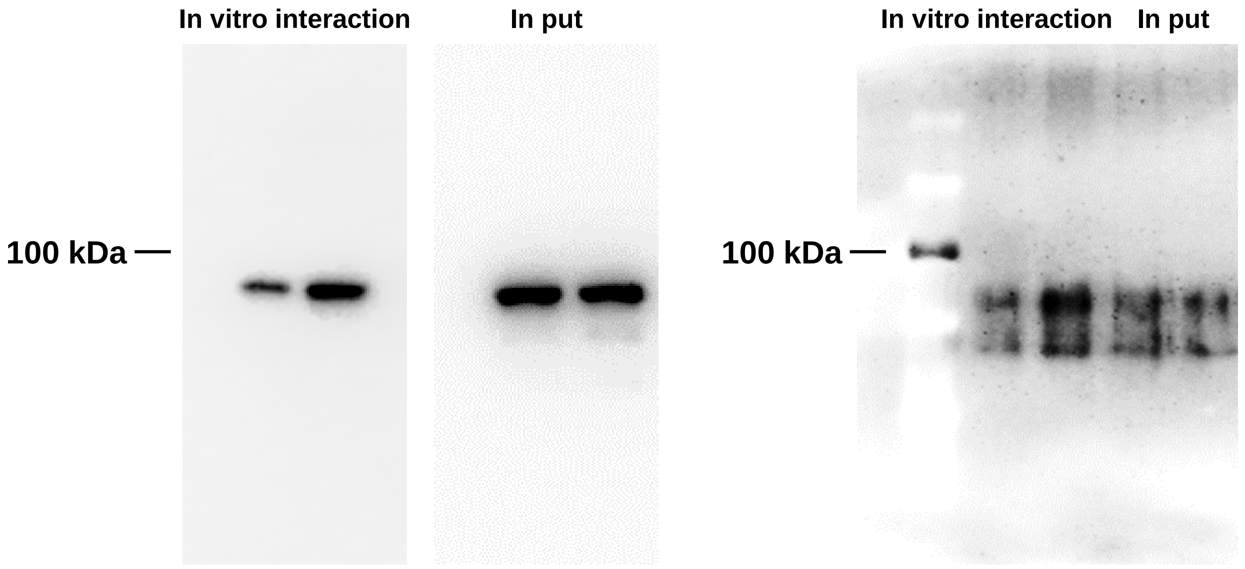

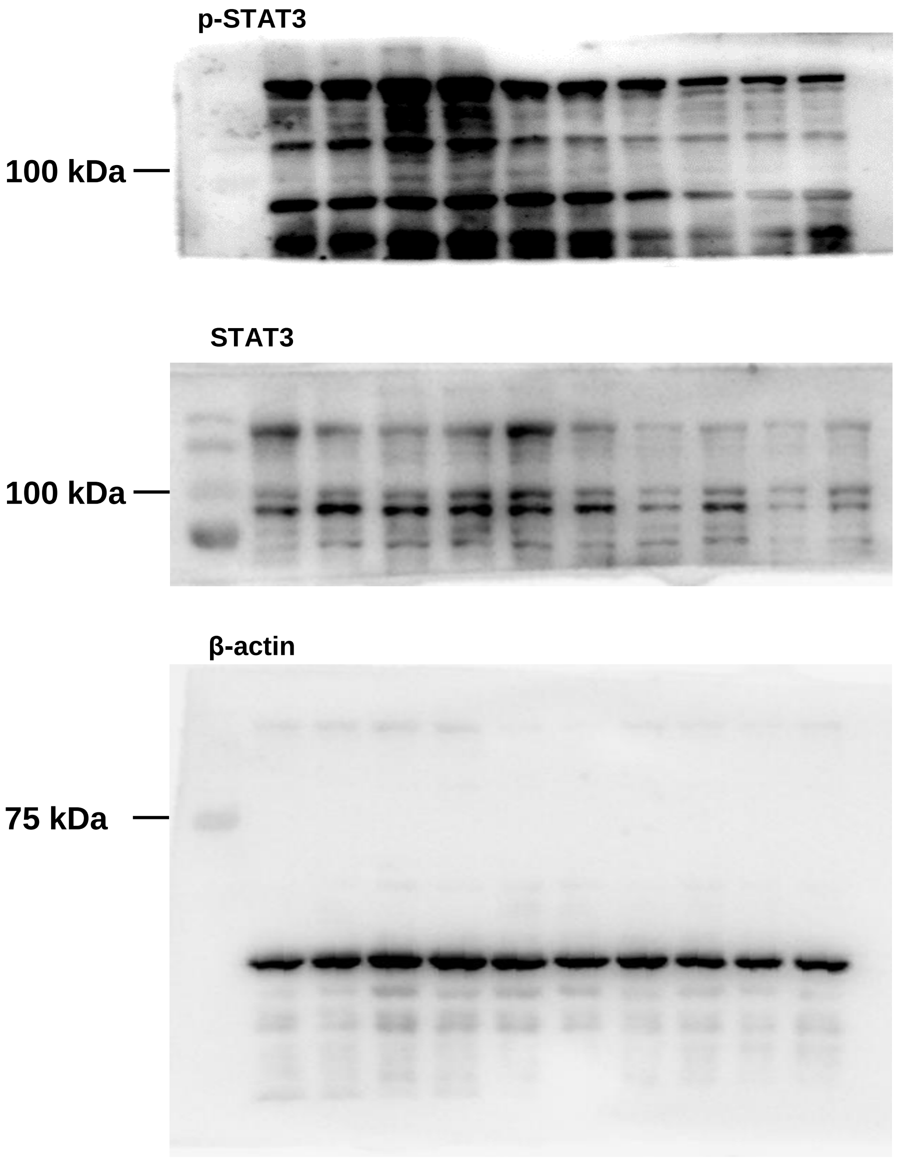

Supplement: Supplementary file 1 — Supplymentary information (Western blot whole blot) [file 41598_2018_32497_MOESM1_ESM.pdf]
